# Development of a graph convolutional neural network model for efficient prediction of protein-ligand binding affinities

**DOI:** 10.1371/journal.pone.0249404

**Published:** 2021-04-08

**Authors:** Jeongtae Son, Dongsup Kim

**Affiliations:** Department of Bio and Brain Engineering, Korea Advanced Institute of Science and Technology, Daejeon, South Korea; Aberystwyth University, UNITED KINGDOM

## Abstract

Prediction of protein-ligand interactions is a critical step during the initial phase of drug discovery. We propose a novel deep-learning-based prediction model based on a graph convolutional neural network, named GraphBAR, for protein-ligand binding affinity. Graph convolutional neural networks reduce the computational time and resources that are normally required by the traditional convolutional neural network models. In this technique, the structure of a protein-ligand complex is represented as a graph of multiple adjacency matrices whose entries are affected by distances, and a feature matrix that describes the molecular properties of the atoms. We evaluated the predictive power of GraphBAR for protein-ligand binding affinities by using PDBbind datasets and proved the efficiency of the graph convolution. Given the computational efficiency of graph convolutional neural networks, we also performed data augmentation to improve the model performance. We found that data augmentation with docking simulation data could improve the prediction accuracy although the improvement seems not to be significant. The high prediction performance and speed of GraphBAR suggest that such networks can serve as valuable tools in drug discovery.

## Introduction

Computational methods, which have been developed and improved in the past decades, have gained increased influence on drug discovery. During the initial phase of the drug discovery process, the prediction of the binding affinity of a candidate ligand for a therapeutic target is an important step. Although many computational techniques have been developed using numerous tools, developing novel drug candidates remains a complicated and difficult task. Conventionally, two main computational approaches, namely ligand-based and structure-based methods, have been proposed to predict protein-ligand binding affinities.

The ligand-based method predicts the binding affinity by analyzing the important properties of the known ligands. Since the structural information of the target protein is not required for the prediction, the ligand-based method can be used when the biological and chemical information of the target protein and its ligands is available. This approach often utilizes molecular fingerprints as the representation of ligand structures, allowing the model to use a fixed-length vector effectively. However, missing structural information hampers accurate prediction of the molecular interaction. Structure-based methods can gather valuable information from the 3D structures of compounds. Since molecular interactions are under the influence of the spatial conformation of the constituent atoms, information derived from the structural features can improve prediction performance. Despite the limited number of available databases of 3D protein structures, structure-based approaches have been widely used to predict protein-ligand interactions.

Recently, advances in deep learning algorithms have helped solve many challenging problems, including recognition of speech, inference of vision, and translation of languages, and its applications have expanded to many other areas. Whereas manually generated features are necessary for conventional machine learning algorithms, deep learning methods automatically extract important features from a dataset. Since deep learning approaches have been proven effective in predicting several biological activities, these approaches have also been used in structure-based methods to predict protein-ligand binding affinities.

AtomNet [1] is a deep learning model that predicts molecular binding by using convolutional neural networks (CNNs). It is known as the first major introduction of the deep learning technology into the prediction of protein-ligand interactions by using 3D protein structure information. AtomNet uses the complex structures of target proteins and small molecules, which are voxelized into a cube of 20Å. To represent the structure of a protein-ligand complex, the environment of the atoms in the 3D structure is encoded into the fixed form of the feature vectors. 3D CNNs are applied to the voxel volumes of AtomNet, and then a binary classification model that classifies the ligands as either active or decoy is developed. Pafnucy [2] is another convolutional neural network model that predicts protein-ligand binding affinities by using 3D CNNs. It tries to solve the regression problem, instead of binary classification problem, that predicts the value of the protein-ligand binding affinity, which enhances its potential for use in drug discovery.

Although the major stream of the deep learning model with 3D structural information is focused on convolutional neural networks [1, 2], this approach is costly and requires too many computational resources. Moreover, 3D convolutional neural network models, which are very sensitive to the orientation of the complex, can be trained by the same structures with many different orientations. This approach may not be efficient, as it requires too much computing time to use 3D convolutional neural networks with large databases to cover the rotational information of the complex structures. To overcome these limitations in the 3D convolution operation, we decided to use the graph convolutional network approach instead of the convolutional neural network for a more effective feature representation of a binding structure.

Graph convolution has been used for improving the performance of the models based on a CNN. Since the molecular structure, typically represented as a string, such as SMILES, can also be easily represented in a graph form, many studies have used graph convolutional networks for deep learning methods. Gomes *et al*. [3] have presented the concept of graph convolution as atom type convolution with the distance matrix and neighbor list. GraphDTA [4] is a model that combines two modules, the graph-based module with SMILES and a 1D convolutional neural network model with protein sequences. DeepAffinity [5] has tried 2D-graphs-form compound representation to overcome the limitation of SMILES strings, but the performance achieved is not substantially different from those of other CNN models. Lim *et al*. [6] have proposed a graph convolutional network model that directly incorporates the 3D structural information of the complex. They have transcribed the 3D structure of a protein-ligand complex into a graph and predicted the drug-target interaction. Torng and Altman [7] have combined the 3D graph representation of a protein pocket structure with the 2D graph representations of ligands from the SMILES. On virtual screening, their model shows better performance than the CNN model and the docking simulation. Although these graph convolutional neural network models have shown potential in predicting protein-ligand interactions, the previous studies have focused on classification of the drug-target interactions, and, to our knowledge, there is no regression model predicting protein-ligand binding affinity values by using 3D structural information with the graph convolutional neural network.

Francoeur *et al*. [8] have found that data augmentation with low quality re-docking data can improve model performance. Despite the caution from the authors that the improvement might have resulted from the expanded data volume, their report shows that data augmentation with structural information improve the predicting power of a model.

In this paper, we propose a novel model, GraphBAR, which uses a graph convolutional network to assess protein-ligand binding affinities. Instead of using a 3D voxelized grid cube, atomic features and environmental information are represented in a graph form, whereby the model can predict binding affinities more efficiently and accurately. The reduced computing power and increased speed from graph convolution enable data augmentation with docking simulation. According to our model, data augmentation can slightly improve the performance of a model in several datasets. Consequently, the data limitation in structure-based approaches can be overcome. Accordingly, GraphBAR provides an alternative approach to use the 3D structural information of protein-ligand complexes.

## Materials and methods

### Graph convolution

Here, we describe the structure of a protein-ligand complex in a graph representation. Graphs can be constructed with nodes and edges, where the nodes retain feature vectors representing their own properties and the edges retain information associated with the connections among nodes to show the graph structure. The inputs of the graph convolution layer are an *N*×*F* feature matrix **F**, where *N* is the number of nodes and *F* is the number of input features, and an *N*×*N* adjacency matrix **A**, which has information regarding the node interactions. The value on the *i*-th row and j-th column in the adjacency matrix **A** is > 0 when the i-*th* and j-*th* nodes are connected, and 0 otherwise. After defining the modified adjacency matrix **Ã** as **A** + **I**, where **I** is an identity matrix, simple matrix multiplication of the feature matrix **F** and the modified adjacency matrix **Ã** by using a dot product in the graph convolution layer (i.e., **Ã ·F**) can sum the feature values of the neighboring nodes and pass it into the center node. After passing the graph convolution layer and a non-linear activation function σ, an output matrix **Fʹ** is produced in the *N*×*F***ʹ** matrix form, where *F***ʹ** denotes the number of output features of the node. Since each graph depicts a different number of nodes, the adjacency matrix should be normalized to stabilize the scale of the feature vectors. We normalized the modified adjacency matrix by using the diagonal node degree matrix of **Ã.** Let **W** be the trainable weight matrix, then the graph convolution can be written as in Eq 1 below.

$$F'=\sigma (D^{-\frac{1}{2}}\tilde{A}D^{-\frac{1}{2}}FW)$$ (1)

The result of graph convolution shows that every node has its own feature vector value. However, to predict the final binding affinity value, we require the representative vector for the entire graph. We found that the graph gather layer can be used to design the model [9]. In the graph gather layer, all values are simply summed up to return graph featurization. The graph features from the graph gather layer are used to predict the binding affinity of the graph at the final stage of the model.

### Dataset

In conventional molecular docking simulations, the structures of ligands are treated as flexible when the structures of proteins are treated as rigid. The PDBbind database [10] is a collection of experimentally measured binding affinity data for protein-ligand complexes. This database was divided into three subsets, namely the general, refined, and core sets. The general set included all the data in the database, even in cases of low quality. The refined set included the collected data with greater quality refinement and improved resolution. The core set was selected from the refined set and consisted of representative data from the clustered refined set data. We prepared several different datasets to determine the data handling of the graph convolutional neural network with different sizes of the data.

The simplest dataset, which was named “dataset 1,” consisted of the PDBbind 2016 refined set. From the refined set, we removed 290 complexes of the core set, which was used as the test set, and 82 overlapping complexes of the PDBbind 2013 core set, which was used as another test set. The remaining complexes were split into two subsets—the training and validation sets for model training. We collected the validation set, which consisted of 10% of the complexes in the refined set, which were randomly selected.

The second dataset, “dataset 2,” was a data-augmented version of dataset 1. Since graph representation uses less computational time than the 3D cube representation, it is possible to use data augmentation to improve model performance. For the data augmentation, we generated the docking structure of the protein-ligand binding. The 3D structure of bound protein and ligand was obtained from a docking simulation performed using the QuickVina2 program (https://qvina.github.io/) [11] with structural information obtained from the PDBbind 2016 dataset. We added docking structures with the highest docking scores for each complex, excluding the complexes whose RMSD was > 3.0. We restricted the number of docking structures up to three per complex to avoid bias from adding too many docking structures with low RMSD for certain complexes, eventually leading to 11,973 structures of 3,316 complexes as the training set, and 1,362 structures of 369 complexes as the validation set.

We built “dataset 3” and “dataset 4” by using the same criteria as used for datasets 1 and 2, except the PDBbind 2016 general set instead of the PDBbind 2016 refined set was used for datasets 3 and 4. We removed the overlapping structures from the PDBbind 2016 core set and the PDBbind 2013 core set from all the complexes of PDBbind 2016, and partitioned these into training and validation sets, with a 9:1 ratio. We curated the validation set from the PDBbind 2016 refined set only, which had qualified structural information, and named this as “dataset 3.” Additional docking simulation results for the complexes of dataset 3 were calculated and applied for the training of the model named “dataset 4.”

With these four datasets, we compared the cases with low numbers of well-refined complexes to the cases with large numbers of disorganized complexes, and aimed to identify whether data augmentation techniques are required for improved efficiency. The total number of complexes included in each data set is reported in **Table 1**.

**Table 1:** Total number of complexes in each data set.

Dataset	training set	validation set
dataset 1	3,319	369
dataset 2	11,973	1,362
dataset 3	11,146	1,280
dataset 4	38,560	4,622

### Graph representation

**Fig 1:**
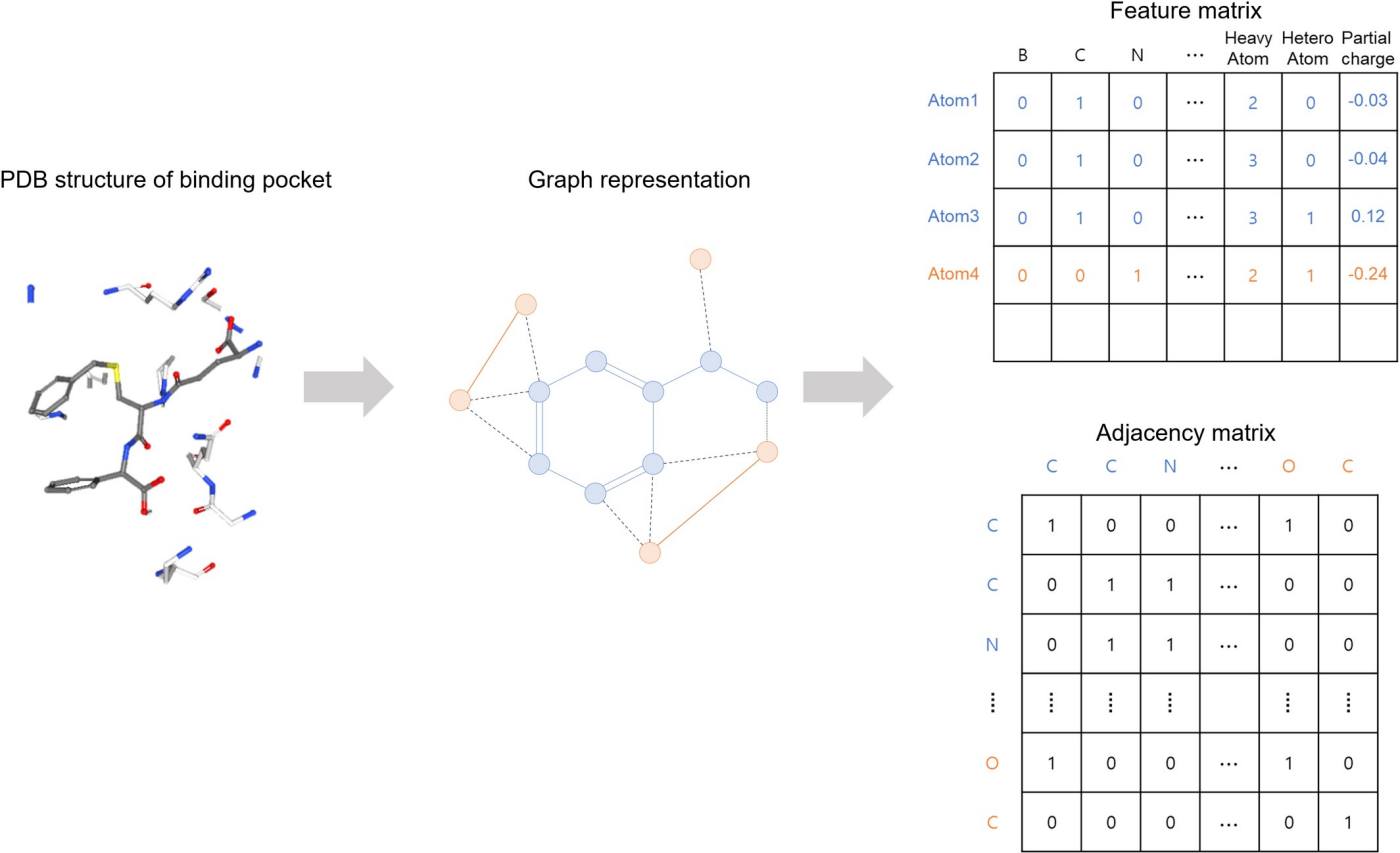
Graph representation of protein-ligand binding structure. The 3D structure of the binding pocket is represented as a graph. The features of the atoms and the features of the atomic interactions are embedded in the feature matrix and adjacency matrix, respectively.

We translated the binding complexes to a collection of graphs. Fig 1 shows the scheme of graph representation with the structural database. We defined the binding site of the complex as the distance from ligand binding. All the atoms in the ligand structure and the protein atoms within a cut-off distance of 4.0 Å from the ligand structure were treated as the nodes in the binding pocket. The binding complex was featured into the fixed size of the feature matrix and the adjacency matrix. Therefore, we filtered out complexes with > 200 nodes at the binding site and used zero-padding to make the size of the matrices equivalent. We adapted the set of features based on those detailed by Stepniewska-Dziubinska *et al*. [2], and all the descriptors were calculated using the Open Babel software (http://openbabel.org/) [12]. Consequently, 13 features were used for the featurization of each atom for GraphBAR (**Table 2**), and they were integrated into a 200×13 matrix to enter the input layer.

**Table 2:** Description of the features.

Features	Description
Atom types	One-hot (B, C, N, O, P, S, Se, Halogen, Metal)
Atom hybridization	Integer (1,2,3)
Heavy-valence	Integer (number of non-hydrogen atoms attached)
Hetero-valence	Integer (number of hetero-atoms attached)
Partial charge	Float

To describe the graph structure of the complex, we constructed the adjacency matrix to embed the information regarding the distances between the atoms at the binding site. Previous research has shown that adjacency matrices embed distance information to reflect intermolecular reactions in float form, since the values in an adjacency matrix increase as the distance between the nodes decreases. However, we have aimed for a model with a simpler calculation and reduced computational time to enable data augmentation. Instead of one or two matrices with the float values, we built several models consisting of different numbers of adjacency matrices (1, 2, 4, and 8) to search for the optimal adjacency matrix approach. Each matrix with *n* adjacency matrices represents the distance between the atoms at intervals of (4/*n*) Å. For example, in the case of the 4-adjacency-matrices model, the **A**_1_ matrix represents the shortest interaction distances between atoms, and the **A**_4_ matrix embeds the longest interaction distances. We limited the representation of the interactions in the adjacency matrix within 4 Å of inter-molecular distance (interaction between the protein and ligand), and 2 Å of intra-molecular distance. Consequently, the matrices covered > 2 Å represented only the inter-molecular interactions. The adjacency matrices were designed as follows:

$$A_{ki,j}=\{\begin{array}{cc} 1 & \mathrm{if}\;\frac{4(k-1)}{n}<d_{i,j}\leq \frac{4k}{n}\\ 0 & \mathrm{otherwise} \end{array}$$ (2)

(k=1,2,3,…n)

where *d*_*i*,*j*_ is the distance between node *i* and node *j*. Using these adjacency matrices, the interactions between the atoms are categorized based on the distances between the atoms, and this categorization allows the model to obtain information from each distance range. Eventually, using multiple matrices with integer numbers allows the model to extract features more efficiently and reduces calculation time.

### Network architecture

**Fig 2:**
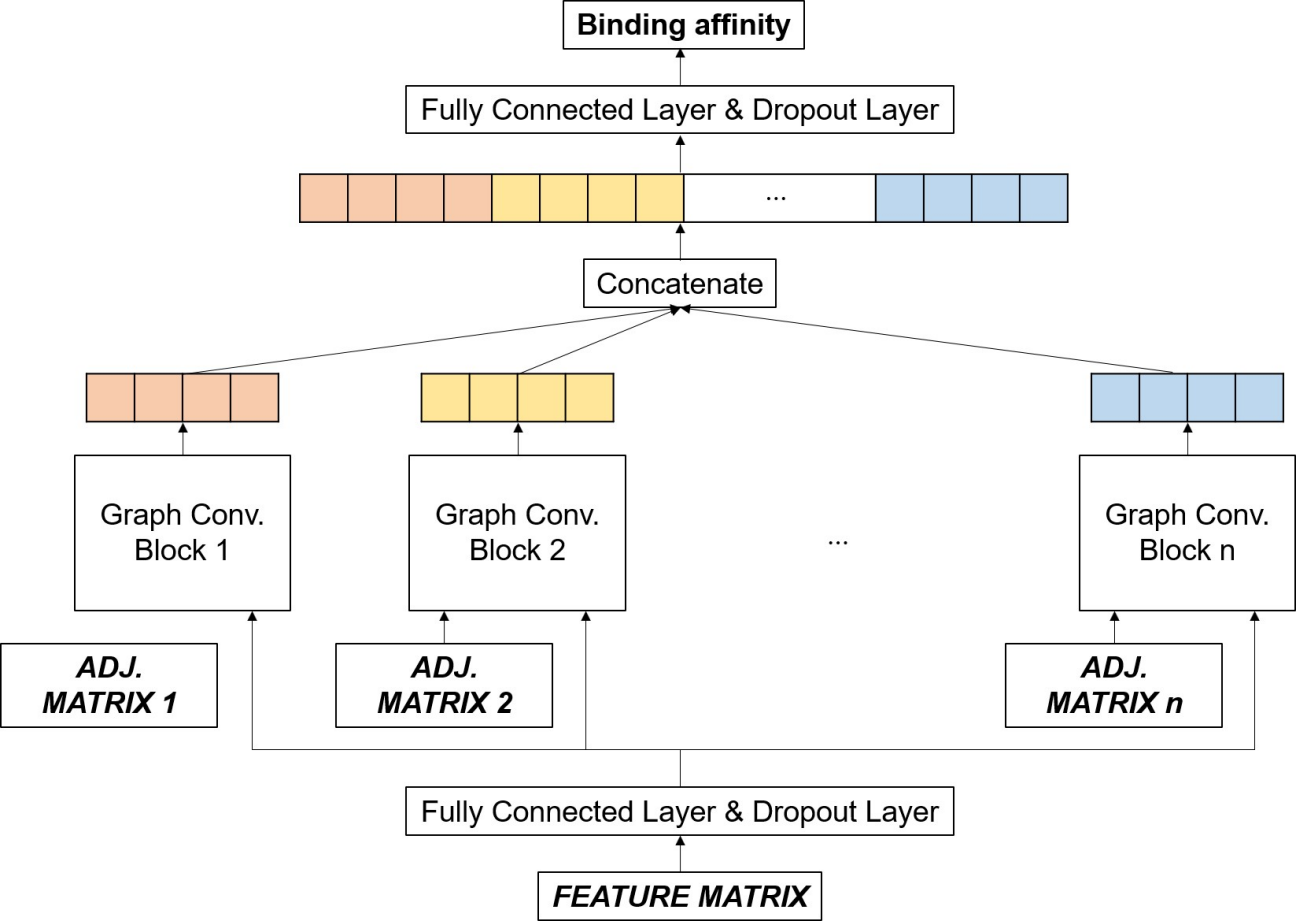
Overview of the GraphBAR architecture. Multiple graph convolution blocks process node features with different adjacency matrices. All the outputs from the graph convolution blocks are concatenated and entered into the fully-connected layer and dropout layer to predict the binding affinity.

**Fig 3:**
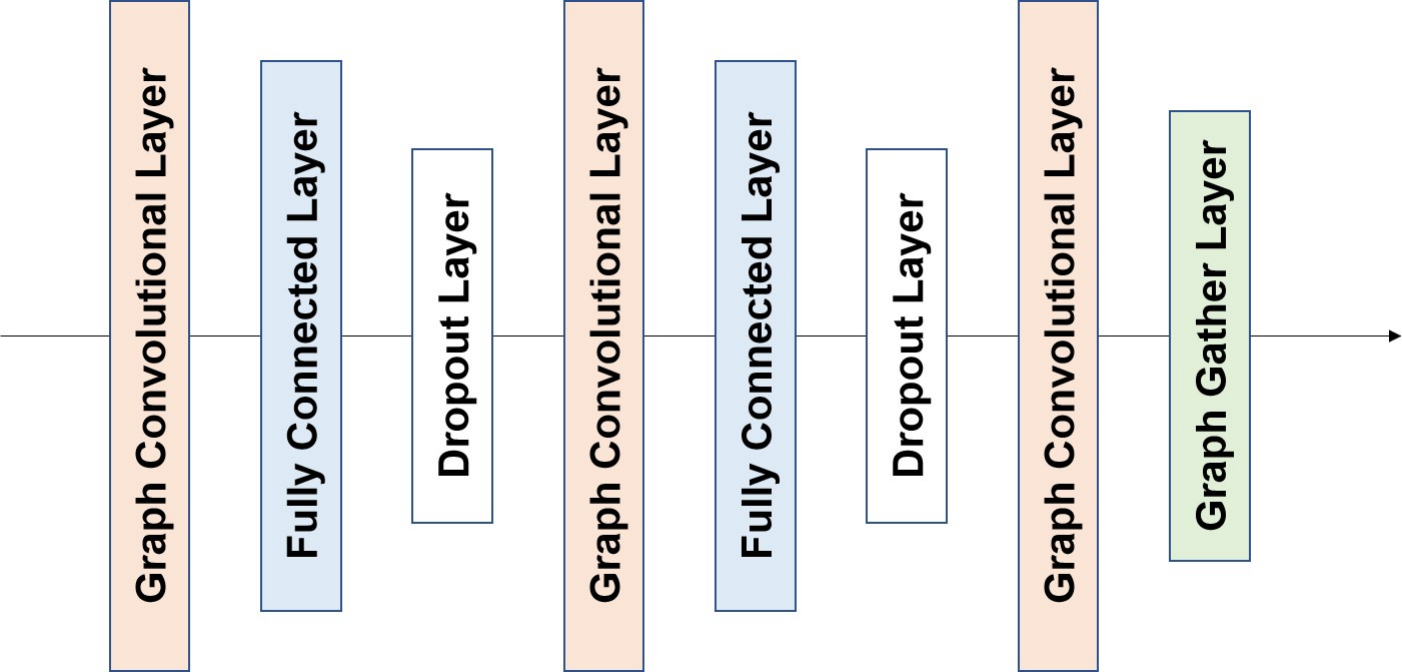
The framework of the graph convolutional block. Each graph convolutional block consists of three graph convolutional layers. The graph convolutional layers are combined with a fully connected layer and a dropout layer, except for the last graph convolutional layer, which is followed by the graph gather layer.

GraphBAR was defined and implemented using TensorFlow v1.12 [13]. The architecture of the proposed model is illustrated in Fig 2. The inputs of the model are one feature matrix and multiple adjacency matrices. First, the input feature matrix was processed by the fully-connected layer and the dropout layer. Features extracted from these layers were fed into the different graph convolution blocks with one of the adjacency matrices. The graph convolution block had three graph convolution layers, composed of a fully-connected layer and a dropout layer between them (Fig 3). The output of the last graph convolution layer was connected to the graph gather layer, which sums all the node feature values to create features representing the entire graph. All the outputs of the graph convolution blocks were concatenated, followed by the fully connected layer with the dropout layer, and output layer for binding affinity prediction.

The model structure and hyperparameters were empirically determined. We searched for possible options to select as the number of graph convolution layers (1 to 5), the number of fully-connected layers in several locations (0 to 3), the dimension of the graph convolution layer (8 to 1024), optimizer type (ADAM, SGD, RMSprop, Momentum), learning rate (0.001 to 1), and dropout rate (0.3 to 0.8). We used 128 neurons for the first and last fully-connected layers, and (128, 128, 16×*n*) neurons for the three fully-connected layers in a graph convolution block with *n*-adjacency matrices. The dimensions of the three graph convolution layers were (128, 128, 32). To train the model, Adam optimizer with 0.001 learning rate was used, and to avoid overfitting, all the dropout layers had a dropout rate of 0.5. Both graph convolution layers and fully-connected layers were implemented with the ReLU activation function, and the mean squared error was used as the loss function. We used the early-stopping algorithm, to stop the training of the model when the monitored validation error exhibited an increasing pattern.

## Results

### Evaluation criteria

To evaluate the performance of the model, the root mean square error (RMSE) and the mean absolute error (MAE) were calculated for prediction error, and the Pearson’s correlation coefficient and the standard deviation (SD) were calculated for correlation between experimentally measured values and predicted values. The SD in this study was defined as described by Li *et al*. [14] as follows:

$$SD=\sqrt{\frac{{\sum \left[y_{i}-(a+bx_{i})\right]}^{2}}{N-1}},$$ (3)

where *x*_*i*_ and *y*_*i*_ are the predicted binding affinity and the experimentally measured binding affinity of the *i*-th complex, respectively, and *a* and *b* are the slope and intercept of the regression line, respectively, between the predicted and actual values.

### Model performance

In this study, we designed a novel model to predict the protein-ligand binding affinity by using a graph convolutional network. We evaluated the model performance for the PDBbind v.2016 core set. Four datasets, which were prepared according to the description in the Dataset section, were evaluated five times, and the mean values with the standard deviations in parentheses are presented in Table 3. We also evaluated the performance of Pafnucy [2] on the same datasets, and compared our results with those obtained using the CNN model. As mentioned in the Graph representation section, we filtered out 111 complexes that had > 200 nodes. It appears that this removal caused the prediction accuracy of Pafnucy slightly lower than the reported accuracy (RMSE 1.42, R 0.78), since the model showed a similar performance to the reported one when we used all the structures. The performance of the model was improved for datasets 2, 3, and 4, indicating that data number is critical for the performance of the model. In particular, the results revealed that when the number of data was low (dataset 1), even simple data augmentation with low quality docking data could improve the performance of the graph convolution model as well as the CNN model. However, when the number of data was high enough, data augmentation with docking simulation did not improve the prediction power (dataset 4). Additionally, the graph convolution model showed a better performance than the CNN model when the number of data was low.

**Table 3:** The performance of GraphBAR on the PDBbind v.2016 core set.

Dataset	Model	RMSE	MAE	SD	R
**dataset 1**	Adj-1	1.609 (0.037)	1.298 (0.029)	1.527 (0.034)	0.712 (0.018)
	Adj-2	1.498 (0.056)	1.202 (0.043)	1.452 (0.062)	0.746 (0.026)
	Adj-4	1.522 (0.057)	1.216 (0.045	1.460 (0.037)	0.742 (0.018)
	Adj-8	1.542 (0.051)	1.241 (0.047)	1.495 (0.037)	0.726 (0.015)
	Pafnucy	1.600 (0.030)	1.304 (0.026)	1.573 (0.029)	0.690 (0.012)
**dataset 2**	Adj-1	1.548 (0.056)	1.253 (0.050)	1.478 (0.046)	0.734 (0.019)
	Adj-2	1.495 (0.031)	1.204 (0.026)	1.440 (0.026)	0.748 (0.011)
	Adj-4	1.535 (0.041)	1.223 (0.043)	1.501 (0.036)	0.724 (0.015)
	Adj-8	1.533 (0.059)	1.233 (0.049)	1.474 (0.045)	0.734 (0.021)
	Pafnucy	1.518 (0.039)	1.193 (0.032)	1.511 (0.022)	0.718 (0.011)
**dataset 3**	Adj-1	1.477(0.043)	1.197 (0.039)	1.437 (0.056)	0.750 (0.024)
	Adj-2	1.441 (0.075)	1.155 (0.066)	1.404 (0.060)	0.764 (0.023)
	Adj-4	1.468 (0.060)	1.182 (0.042)	1.406 (0.052)	0.764 (0.021)
	Adj-8	1.502 (0.052)	1.202 (0.052)	1.455 (0.058)	0.742 (0.027)
	Pafnucy	1.465 (0.033)	1.164 (0.026)	1.441 (0.043)	0.748 (0.018)
**dataset 4**	Adj-1	1.490 (0.034)	1.210 (0.030)	1.418 (0.030)	0.760 (0.012)
	Adj-2	1.413 (0.038)	1.144 (0.033)	1.371 (0.048)	0.778 (0.016)
	Adj-4	1.467 (0.046)	1.190 (0.036)	1.405 (0.035)	0.764 (0.015)
	Adj-8	1.512 (0.040)	1.217 (0.030)	1.455 (0.026)	0.742 (0.011)
	Pafnucy	1.511 (0.042)	1.210 (0.040)	1.503 (0.044)	0.722 (0.018)

We compared our results on the PDBbind v. 2013 core set with the results reported by other groups–X-Score by Li *et al*. [15], which shows the best performance, and Pafnucy [2] (Table 4). We chose the model with two adjacency matrices as the representative model since this model showed the best performance on the PDBbind 2016 database. The results indicated that our graph convolutional model showed competitive performance with the scoring function model or CNN model. However, the data augmentation with docking simulation was not improved for small and large database on PDBbind v.2013 core set. We infer that this gap stemmed from the composition of the data sets. Since the PDBbind v.2016 core set was selected from the v.2016 refined set as the representative complexes, they shared certain patterns regarding properties. In contrast, the PDBbind v.2013 core set contained a part of the non-refined data from the v.2016 refined set; this pattern was not well arranged like data newly introduced to a trained model. Despite the modest success with the low number of data in the PDBbind 2016 database, the data augmentation with the docking simulation had a limitation for the situation as the disparity between test data and trained data exists.

**Table 4:** The performance of GraphBAR with the PDBbind v.2013 core set.

Dataset	RMSE	MAE	SD	R
**dataset1**	1.694 (0.065)	1.368 (0.056)	1.680 (0.068)	0.662 (0.033)
**dataset2**	1.688 (0.032)	1.367 (0.019)	1.669 (0.030)	0.670 (0.019)
**dataset3**	1.616 (0.030)	1.322 (0.040)	1.597 (0.025)	0.704 (0.011)
**dataset4**	1.636 (0.035)	1.317 (0.031)	1.601 (0.041)	0.704 (0.018)
**X-Score**	-	-	1.78	0.61
**Pafnucy**	1.62	1.32	1.61	0.70

We also tested our model on the other independent data set, to measure generalization of our model. CSAR NRC-HiQ data set [16] provided by CSAR (csardock.org) is another widely known data, which involves two subsets of 176 and 167 complexes respectively. The complexes overlapped with our training set were excluded resulting only 51 and 36 complexes were used for testing. We compared GraphBAR model with two-adjacency matrices and Pafnucy which is trained with the dataset3 to measure the performance on CSAR NRC-HiQ data set (Tables 5 and 6). GraphBAR outperforms on the CSAR NRC-HiQ set 1, whereas the data augmentation did not improve the performance of the model as the test on PDBbind 2013 core set. On the CSAR NRC-HiQ set 2, GraphBAR with the data augmentation shows similar performance to the CNN model. Both methods show reasonable errors to predict the binding affinity on a new dataset, and therefore, GraphBAR could be used more generally.

**Table 5:** The performance of GraphBAR with the CSAR NRC-HiQ set 1 (51 complexes).

Dataset	RMSE	MAE	SD	R
**dataset1**	1.741 (0.046)	1.368 (0.059)	1.588 (0.057)	0.732 (0.023)
**dataset2**	1.709 (0.073)	1.373 (0.049)	1.651 (0.113)	0.704 (0.049)
**dataset3**	1.599 (0.108)	1.218 (0.105)	1.569 (0.123)	0.738 (0.047)
**dataset4**	1.592 (0.092)	1.280 (0.071)	1.534 (0.058)	0.754 (0.023)
**Pafnucy**	1.707 (0.100)	1.338 (0.092)	1.612 (0.069)	0.722 (0.025)

**Table 6:** The performance of GraphBAR with the CSAR NRC-HiQ set 2 (36 complexes).

Dataset	RMSE	MAE	SD	R
**dataset1**	1.873 (0.198)	1.414 (0.114)	1.756 (0.154)	0.456 (0.145)
**dataset2**	1.558 (0.040)	1.182 (0.074)	1.510 (0.075)	0.652 (0.041)
**dataset3**	1.715 (0.103)	1.349 (0.035)	1.683 (0.068)	0.538 (0.052)
**dataset4**	1.584 (0.104)	1.273 (0.073)	1.576 (0.129)	0.610 (0.083)
**Pafnucy**	1.554 (0.034)	1.256 (0.039)	1.540 (0.043)	0.636 (0.027)

On the whole predictions, the data augmentation methods maintain the performance of the model, and improve the performance in several cases for the graph convolutional model. Though the effect of the data augmentation is not significant, we hope that this approach could be a new suggestion for other areas suffering from low amount of data.

### Efficient calculation with Graph convolutional neural network

We measured the computational time for training with the PDBbind database to assess whether GraphBAR could be trained more efficiently with the reduced computational time than the CNN model. We also compared our multiple-adjacency-matrices approach model with the integer values relative to the model using the adjacency matrix with the float values. We measured the computational time for training by summing all calculation times for each training and validation epoch. The mean values and the standard deviations from four runs per task are reported in Table 7. As expected, all the graph convolution models with integer values required a shorter training time than the model with float values or the CNN model. According to the results, the training with GraphBAR with two adjacency matrices required roughly 5% of the training time required with the CNN model. We also measured the inference time for both models, however, the inference time is too short to discuss about the computational efficiency. We provide the inference time and the data preparation time in S3 Table. These results indicate that the graph convolution method can decrease the computing time while maintaining a competitive performance in comparison with other models, such as Pafnucy [2]. Thus, GraphBAR is much more amenable to the data augmentation method than other models.

**Table 7:** The training time of the models with the PDBbind 2016 database.

Dataset	Model^a^					
	Adj-float	Adj-1	Adj-2	Adj-4	Adj-8	Pafnucy
**dataset1**	541.22 (3.33)	4.84 (0.02)	10.87 (0.06)	27.78 (0.10)	40.30 (0.18)	161.83 (0.04)
**dataset2**	2238.41 (11.64)	16.41 (0.05)	30.27 (0.02)	70.87 (0.19)	105.94 (0.52)	681.87 (0.12)
**dataset3**	2952.48 (10.20)	19.22 (0.03)	39.93 (0.16)	69.36 (0.30)	157.68 (0.29)	557.76 (0.13)
**dataset4**	8065.14 (20.47)	41.52 (0.11)	97.19 (0.27)	192.44 (0.41)	420.81 (1.31)	2236.35 (0.76)

^a^Computing time were measured in minute scale.

## Conclusions

In this paper, we introduced a novel prediction model for protein-ligand binding affinity, which can be used for drug discovery. Instead of the conventionally used CNN model, the graph convolution approach of GraphBAR offers several advantages. The model can predict the binding affinity of a protein-ligand complex more efficiently, regarding computing efficiency and calculation time, while displaying a competitive performance compared with recently published prediction models. Moreover, the performance of GraphBAR can be further improved via data augmentation.

A graph convolution model that uses 3D structural information underlies the aim of this study. Our results show that the graph convolutional neural network can successfully predict the binding affinity with acceptable accuracy. A graph convolutional model ensures that the model can handle 3D structural information easily. This model can dramatically reduce the calculation time while allowing data augmentation.

A limitation of this study is the limited data regarding structure-based deep learning approaches to predict protein-ligand binding affinities. Available databases that contain detailed structural information regarding both proteins and ligands are not abundant, and the amount of data in the available databases is considerably low. Despite the promising features of the 3D structural information of a protein-ligand complex, this limitation impedes efficient prediction. We employed the data augmentation method, which is difficult to apply to the CNN model due to computational cost. The effect of data augmentation with docking simulation was verified using small datasets, indicating the potential of GraphBAR for data augmentation applications alongside computational efficiency.

Moreover, we tried to improve the performance of GraphBAR via supporting methods, such as graph attention network [17] and capsule network [18]. These layers normally employ softmax functions and are optimized for classification prediction. As a result, a new model that uses graph attention networks instead of graph convolutional layers, or a capsule network instead of graph gather layers, showed a poor performance (S1 Table). These approaches may improve drug-target interaction prediction as a binary classification, but did not work well in binding affinity prediction.

For data augmentation, we used the docking results of the PDBbind database. The results measuring the training time indicate that the graph convolutional neural network enables data augmentation due to its computational efficiency. In addition, the results on several test datasets indicate that data augmentation with docking simulation slightly improves the prediction accuracy although the performance gain seems not to be significantly high, and give a flexibility to the model for prediction on newly introduced data set. We hope that another data augmentation method could be found to improve the performance of the model in such situations. Further, with the graph convolutional approach, the model can overcome the limited availability of structural information regarding protein-ligand binding.

GraphBAR can provide the values for the predicted binding affinity of a protein-ligand complex. Therefore, GraphBAR can be used for in silico screening to identify specific targets or target prediction for specific chemicals. The model can be introduced into drug discovery pipelines and further research endeavors, such as the prediction of ADMET properties. Moreover, the reduction in the computing resources needed ensure that the model can be used during the early stages of drug discovery, which would be interesting in the computational biology field.

## Supporting information

S1 Fig Distribution of the predicted values for dataset 1 best models on the PDBbind v.2013 core set with 2-adjacency matrix model. (TIF)

S2 Fig Distribution of the predicted values for dataset 2 best models on the PDBbind v.2013 core set with 2-adjacency matrix model. (TIF)

S3 Fig Distribution of the predicted values for dataset 3 best models on the PDBbind v.2013 core set. (TIF)

S4 Fig Distribution of the predicted values for dataset 4 best models on the PDBbind v.2013 core set with 2-adjacency matrix model. (TIF)

S1 Table Model performance with graph attention network and capsule network based on a dataset with a 4-adjacency matrix model. The graph attention model used a graph attention network instead of a graph convolution network. The Capsule network model used a capsule network instead of a graph gather layer.
(PDF)

S2 Table Best performance of each model. The best performance of each model among 5 evaluations with the PDBbind v.2016 and 2013 core sets.
(PDF)

S3 Table The inference time of the models with the PDBbind 2016 core set and the data preparation time of the models with the CSAR NRC-HiQ set. (PDF)
