# Supplementary figures and images for: Development of a graph convolutional neural network model for efficient prediction of protein-ligand binding affinities
Source: PLoS One. 2021 Apr 8;16(4):e0249404. doi: 10.1371/journal.pone.0249404 (PMC8031450; doi:10.1371/journal.pone.0249404)

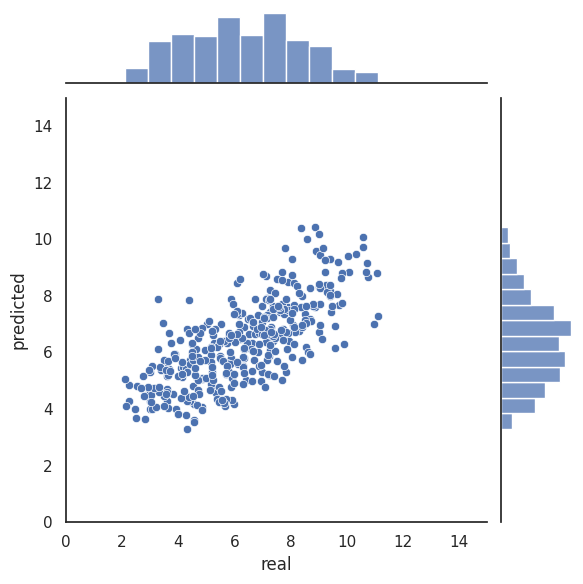

Supplement: S1 Fig — (TIF) [file pone.0249404.s001.tif]

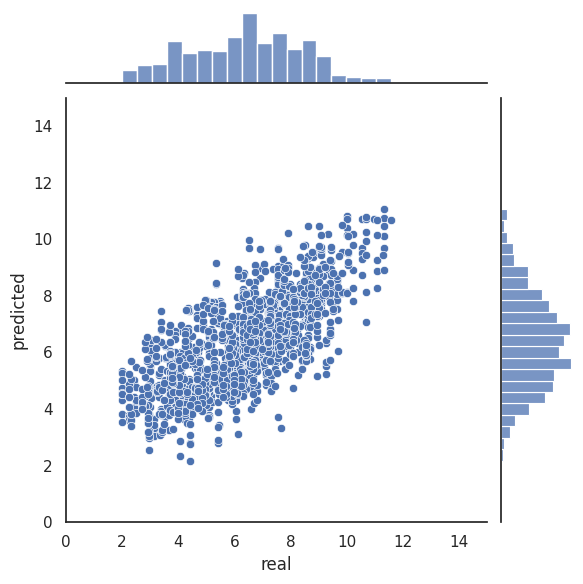

Supplement: S2 Fig — (TIF) [file pone.0249404.s002.tif]

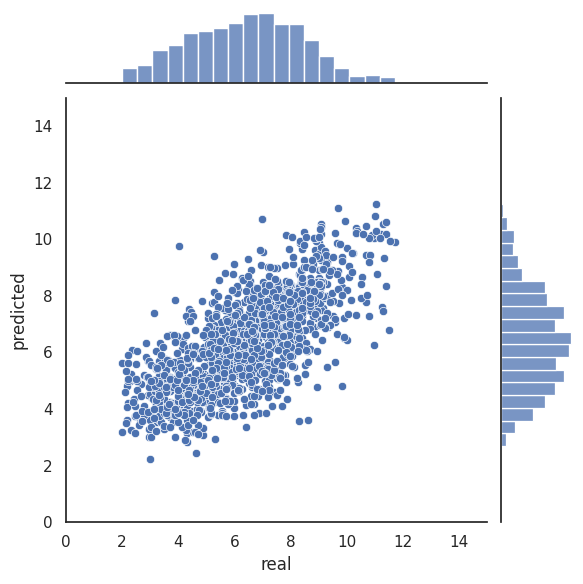

Supplement: S3 Fig — (TIF) [file pone.0249404.s003.tif]

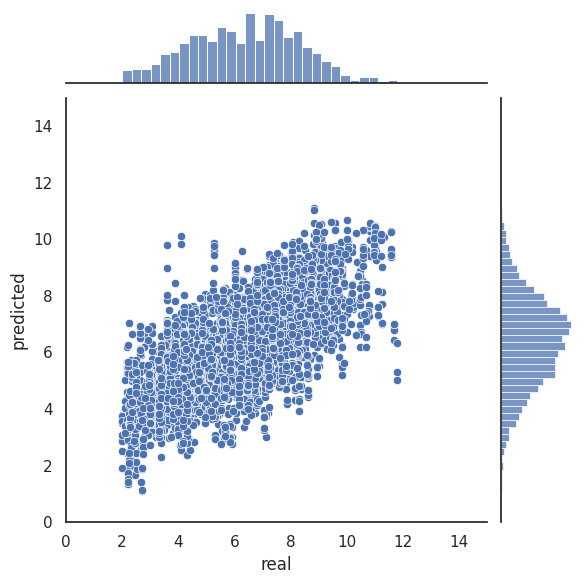

Supplement: S4 Fig — (TIF) [file pone.0249404.s004.tif]
