# Supplementary material for: Development of a graph convolutional neural network model for efficient prediction of protein-ligand binding affinities
Source: PLoS One. 2021 Apr 8;16(4):e0249404. doi: 10.1371/journal.pone.0249404 (PMC8031450; doi:10.1371/journal.pone.0249404)
Supplement: S1 Table — The graph attention model used a graph attention network instead of a graph convolution network. The Capsule network model used a capsule network instead of a graph gather layer. (PDF) [file pone.0249404.s005.pdf]

Table S1a. Model performance with graph attention network based on dataset3

Test data	RMSE	MAE	SD	R
**PDBbind v.2016 core set**	1.7594	1.414	1.7226	0.61
**PDBbind v.2013 core set**	1.848	1.5282	1.8062	0.598

Graph attention model used graph attention network instead of graph convolution network.

Table S1b. Model performance with capsule network based on dataset3

Test data	RMSE	MAE	SD	R
**PDBbind v.2016 core set**	1.7562	1.4214	1.7206	0.612
**PDBbind v.2013 core set**	1.8338	1.5244	1.796	0.604

Capsule network model used capsule network instead of graph gather layer.
