# Supplementary material for: Development of a graph convolutional neural network model for efficient prediction of protein-ligand binding affinities
Source: PLoS One. 2021 Apr 8;16(4):e0249404. doi: 10.1371/journal.pone.0249404 (PMC8031450; doi:10.1371/journal.pone.0249404)
Supplement: S2 Table — The best performance of each model among 5 evaluations with the PDBbind v.2016 and 2013 core sets. (PDF) [file pone.0249404.s006.pdf]

Table S2a. Best performance of each model on PDBbind v.2016 core set

Test data	RMSE	MAE	SD	R
**dataset1**	1.43	1.14	1.40	0.77
**dataset2**	1.45	1.17	1.43	0.75
**dataset3**	1.40	1.10	1.35	0.78
**dataset4**	1.37	1.09	1.36	0.78

Each models's best performance among 10 evaluatoins on PDBbind v.2016 core set.

Table S2b. Best performance of each model on PDBbind v.2013 core set

Test data	RMSE	MAE	SD	R
**dataset1**	1.61	1.27	1.61	0.70
**dataset2**	1.63	1.33	1.61	0.70
**dataset3**	1.47	1.19	1.45	0.77
**dataset4**	1.48	1.22	1.47	0.76

Each models's best performance among 10 evaluatoins on PDBbind v.2013 core set.
