# Supplementary material for: Development of a graph convolutional neural network model for efficient prediction of protein-ligand binding affinities
Source: PLoS One. 2021 Apr 8;16(4):e0249404. doi: 10.1371/journal.pone.0249404 (PMC8031450; doi:10.1371/journal.pone.0249404)
Supplement: S3 Table — (PDF) [file pone.0249404.s007.pdf]

Table S3a. The inference time of the models with the PDBbind 2016 core set (290 complexes)

	Model[a]					
	**Adj-float**	**Adj-1**	**Adj-2**	**Adj-4**	**Adj-8**	**Pafnucy**
**dataset1**	58.65 (0.09)	0.80 (0.15)	1.61 (0.32)	2.68 (0.19)	5.45 (0.52)	2.14 (0.04)
**dataset2**	58.63 (0.19)	0.94 (0.06)	1.36 (0.14)	3.09 (0.36)	5.59 (0.18)	2.13 (0.05)
**dataset3**	58.80 (0.13)	0.92 (0.07)	1.42 (0.18)	3.02 (0.21)	5.55 (0.52)	2.18 (0.05)
**dataset4**	58.55 (0.11)	0.85 (0.04)	1.62 (0.17)	3.09 (0.19)	6.00 (0.85)	2.19 (0.07)

[a]Computing time were measured in second scale.

Table S3b. The data preparation time of the models with the CSAR NRC-HiQ set (51 and 36 complexes)

	**GraphBAR**	**Pafnucy**
CSAR NRC-HiQ set 1	26.65 (0.08)	61.26 (0.24)
CSAR NRC-HiQ set 2	10.77 (0.07)	17.51 (0.11)

Computing time were measured in second scale.
